# Successful Ultra-Minimally Invasive Endoscopic Intrarenal Surgery for 2-Year-Old Boy with Bilateral Cystine Kidney Stones Over 2 cm

**DOI:** 10.1089/cren.2018.0042

**Published:** 2018-07-01

**Authors:** Takaaki Inoue, Masato Watanabe, Seiji Shimada, Hidefumi Kinoshita, Tadashi Matsuda

**Affiliations:** Department of Urology, Kansai Medical University, Osaka, Japan.

**Keywords:** pediatric kidney stones, cystine stones, ultra-minimally invasive endoscopic intrarenal surgery

## Abstract

***Background:*** Treatment of upper urinary tract stones measuring >2 cm in children aged <3 years is challenging. Although adult-sized instruments are usually available, in pediatric populations such instruments seem unreasonable and unfit for children with small kidneys and narrow ureters. We use ultra-miniaturized endoscopes and instruments to reduce the damage to normal tissues in pediatric patients.

***Case Presentation:*** We treated a 2-year-old boy with >2-cm bilateral cystine kidney stones. We decided to perform retrograde intrarenal surgery using an ultrathin (4.5F) semi-rigid ureteroscope for the right kidney stone (2.0 × 1.2 cm) in the lithotomy position and super ultra-minimally invasive endoscopy combined with intrarenal surgery with a percutaneous 8.5F to 9.5F tract sheath for the left kidney stone (3.5 × 2.4 cm) under the Barts modified Valdivia position. These procedures were successful for the bilateral kidney stones. Postoperatively, the patient was stone-free without major complications.

***Conclusion:*** We believe that ultra-minimally invasive endoscopic intrarenal surgery is safe and efficient in pediatric patients. Furthermore, the Barts modified Valdivia position was safely utilized in our 2-year-old patient with multiple large kidney stones.

## Introduction

In pediatric patients, extracorporeal shockwave lithotripsy (SWL) under general anesthesia is usually the first treatment for kidney and proximal ureteral stones of <2 cm. Conversely, percutaneous nephrolithotomy (PCNL) is the standard treatment for kidney stones of >2 cm and is performed using adult instruments including a nephroscope and 24F tract sheath. However, since the first case of stone treatment using ureteroscopy in a pediatric patient was reported in 1977, endourologic surgery using ureteroscopy and nephroscopy has become miniaturized along with technological advancements. Therefore, stone treatment has been changing in the pediatric field.^1^

We herein describe a 2-year-old boy with bilateral cystine kidney stones of >2 cm. We performed ultra-minimally invasive endoscopic intrarenal surgery using an ultrathin (4.5F) semi-rigid ureteroscope in the right kidney without prestenting and super ultra-minimally invasive endoscopy combined with intrarenal surgery (super ultra-mini ECIRS) with an 8.5F to 9.5F tract sheath in the left kidney.

## Case Report

**FIG. 1.**
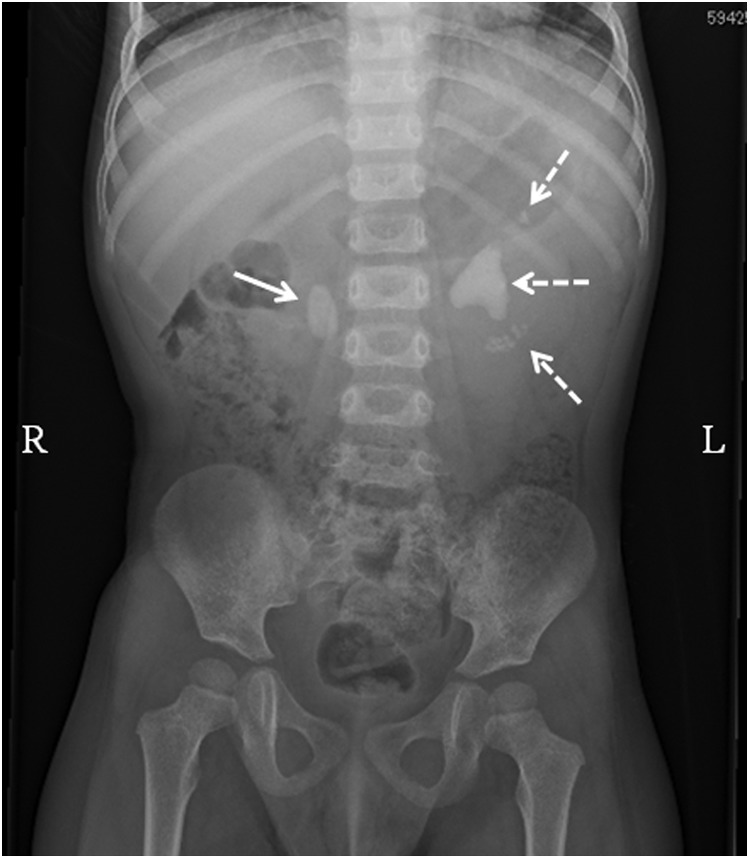
KUB radiograph in our 2-year-old patient. The *arrow* indicates the stone in the pyeloureteral junction of the right kidney. The *dotted arrow* indicates the large kidney stone with multiple stones in the upper and lower poles. KUB, kidney, ureter, and bladder.

We herein describe our experience with a pediatric patient with bilateral large cystine kidney stones. The boy was 2 years old (height, 90.5 cm; weight, 12.8 kg; body mass index, 15.4 kg/m^2^) and had a medical history of a bladder stone 6 months previously; composition analysis revealed a cystine stone. Although bilateral kidney stones were indicated by an imaging study at that time, they were monitored by observation while the patient was prescribed oral medication (tiopronin, 300 mg/day) at the previous hospital. Upon his first consultation at our hospital, his only complaint was right flank pain. Imaging studies including kidney, ureter, and bladder (KUB) radiographs; ultrasonography (US); and computed tomography revealed a large stone measuring 2.0 × 1.2 cm (1043 Hounsfield units) in his right kidney at the pyeloureteral junction with mild hydronephrosis and a partial staghorn stone measuring 3.5 × 2.4 cm (1102 Hounsfield units), with multiple smaller stones in the left kidney (Fig. 1). We developed a strategy of minimally invasive treatment with miniature devices to reduce the patient's operative risk.

**FIG. 2.**
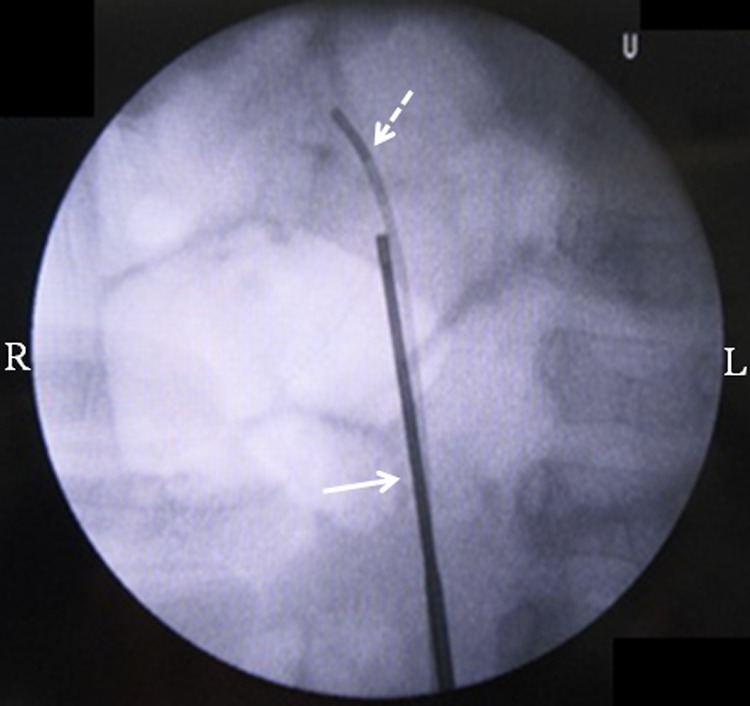
Fluoroscopic image obtained during the procedure using a 4.5F semi-rigid ureteroscope for placement of the ureteral catheter. The *arrow* indicates the 4.5F semi-rigid ureteroscope. The *dotted arrow* indicates the ureteral catheter.

Initially, we planned to perform retrograde intrarenal surgery (RIRS) using a 4.5F ultrathin semi-rigid ureteroscope without preoperative ureteral stenting to treat the right kidney stone in the first procedure. We were prepared to use a flexible ureteroscope (URF-P6; Olympus, Tokyo, Japan) through a 9.5F to 11.5F access sheath if necessary. First, we inserted 0.035-inch guidewire into the right ureteral orifice and then placed the ultrathin semi-rigid ureteroscope over the guidewire while the patient was in the lithotomy position under general anesthesia. The ultrathin semi-rigid ureteroscope was smoothly advanced without resistance and reached the region below the target stone in the pyeloureteral junction. Additionally, we placed a 5F ureteral catheter to decrease the intrarenal pressure because of having some space in the ureter (Fig. 2). We disintegrated the stone using a 272-μm holmium laser (Litho; Rocamed, Monaco, France) in dusting mode (long-pulse mode, 0.8–1.0 J × 10–20 Hz). As stone disintegration progressed, the stone was pushed back to the upper pole; however, we successfully completed disintegration of the stone. Finally, we directly inserted the URF-P6 in the right kidney without an access sheath, extracted some fragments, and placed a 4.8F double-J ureteral stent with no urethral catheter. The operative time was 105 minutes, and the stone components were 95% cystine. The patient had no postoperative complications and was discharged on postoperative day 3. The ureteral stent was removed in an outpatient procedure at 1 week after RIRS. At 1 month postoperatively, US and KUB radiographs revealed no residual fragments or hydronephrosis.

**FIG. 3.**
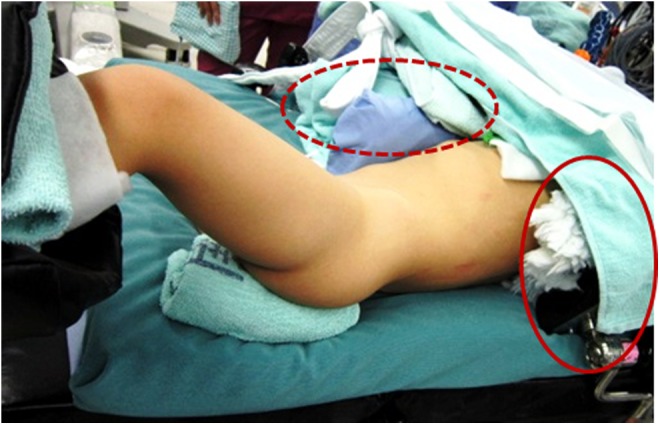
Image of the Barts modified Valdivia position. The *circle* shows the pad used to fix the patient's shoulder. The *dotted circle* shows the thick cushion used to prevent movement toward the median.

**FIG. 4.**
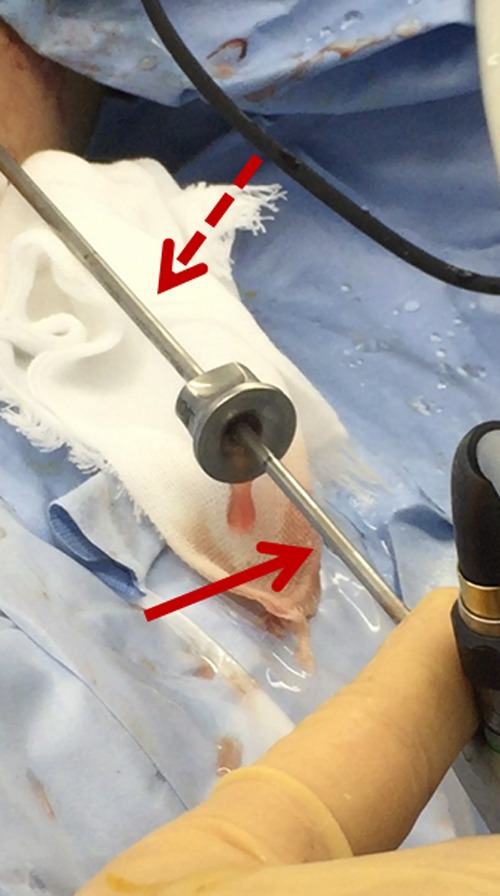
Image of super ultra-mini percutaneous nephrolithotomy. The *arrow* indicates the 6F nephroscope. The *dotted arrow* indicates the 8.5F to 9.8F tract sheath.

Three months later, we treated the partial staghorn stone with multiple caliceal stones in the left kidney. We decided to carry out super ultra-mini ECIRS with simultaneous performance of both RIRS and super ultra-minimally invasive PCNL (super ultra-mini PCNL) with an 8.5F to 9.5F percutaneous tract sheath. The patient was oriented in the Bart's modified Valdivia position under general anesthesia, which is characterized by a lateral position with the upper body elevated at a 45° angle and the lower body in the lithotomy position (Fig. 3).^2^ First, we accessed the left ureter and kidney in a retrograde manner using an ultrathin semi-rigid ureteroscope without resistance and placed a 9.5F to 11.5F access sheath in the upper ureter. A flexible ureteroscope (URF-P6) was inserted through the access sheath, but it could not reach the puncture site in the calyx because of larger stones occupying the renal pelvis. Therefore, we established hydronephrosis and used a 21-gauge needle to puncture the lower calyx under ultrasound guidance with an enhanced vascular flow imaging technique (wideband Doppler method) to avoid major vessels (ARIETTA; Hitachi Aloka Medical, Tokyo, Japan).^3^ We subsequently created the 8.5F to 9.5F tract sheath using a metal one-step dilator under fluoroscopy. We then inserted a 6F miniaturized nephroscope (minimally invasive PCNL generation of nephroscopes; Karl Storz, Tuttlingen, Germany) through an 8.5F to 9.5F tract sheath (Fig. 4). For stone disintegration, a 365-μm holmium laser fiber (Litho; Rocamed) was used for RIRS and super ultra-mini PCNL. We disintegrated the stones using dusting mode (long-pulse mode, 0.8–1.0 J × 10–20 Hz). If percutaneous access to the calyx failed, RIRS was used to help disintegrate and displace the stone. Almost all small fragments were flushed out through the tract sheath or extracted using a nitinol basket. Finally, we placed a 4.8F ureteral stent and 8F nephrostomy tube. The operative time was 185 minutes, and the stone components were 95% cystine. Although the patient developed a mild fever (<39°C) for 2 days postoperatively, he developed no other complications such as visceral injury or the need for transfusion. The nephrostomy tube was removed at postoperative day 3 due to improve postoperative fever. The left ureteral stent was removed on postoperative day 7 and the patient was discharged. At 1 month postoperatively, US and KUB radiographs showed no residual fragments or hydronephrosis.

## Discussion

In the present case, we successfully used ultra-minimally invasive endoscopic instruments for a 2-year-old boy with bilateral large cystine kidney stones.

The incidence of urolithiasis in the pediatric population has been steadily increasing during the past two decades. According to the European Association of Urology/European Society for Pediatric Urology guidelines, SWL is still the first-line treatment for many cases of upper urinary stones in children. The reported stone-free rates after SWL for kidney and ureteral stones are 59.2% to 83.4% and 58.4% to 94.8%, respectively; however, retreatment is needed in almost all cases. Furthermore, these treatments are performed under general anesthesia. Additionally, SWL has the disadvantage of a low stone-free rate for large kidney stones of >2 cm.^4^ Conversely, technological developments have resulted in dramatic miniaturization of ureteroscopes, nephroscopes, and other instruments used in the upper urinary tract. These advancements may offer advantages in pediatric stone treatment. Atar et al. revealed a superior stone-free rate using a 4.5F versus a 7.5F semi-rigid ureteroscope for pediatric ureteral stones in preschool-age children (92.6% *vs* 78.6%, respectively). Moreover, although passive dilation using prestenting was needed in 16.6% of patients in the 7.5F ureteroscope group, no patients (0.0%) in the 4.5F group required passive dilation using prestenting.^5^ In the present case, we obtained smooth access to the stone site using a 4.5F ureteroscope without prestenting and successfully disintegrated the target stone with the dusting mode of a holmium laser. Current laser systems have undergone great improvements. In particular, the changing pulse duration of the holmium laser provides many advantages including less stone retropulsion, less retrieval of fragments, and less technical difficulty. In pediatric patients with large kidney stones, retrieval of fragments using a flexible ureteroscope during RIRS is quite difficult because of children's narrow ureters. Therefore, the dusting technique is very useful in this situation. In the present case, although we extracted as many small fragments as possible to attain a stone-free status, almost all fragments of the disintegrated stone spontaneously passed during the postoperative period. Furthermore, we performed super ultra-mini ECIRS for the left large kidney stones. To our knowledge, this is the first report of a simultaneous retrograde and antegrade approach under the Barts modified Valdivia position in a 2-year-old patient. This Barts modified Valdivia position was first described for adult patients in 2008.^2^ Patients are placed in the lithotomy position with the pelvis tilted at 45° and supported by a foam wedge, while the torso is twisted to the contralateral side with the shoulders perpendicular to the operating table. However, it is problematic to move a pediatric patient's body to the median while performing needle puncture and percutaneous procedures with a nephroscope because of the patient's smaller body size and lower weight. Therefore, we fixed our patient's body by placing a thick cushion on the opposite side. Consequently, the procedure was carried out safely. Additionally, we used wideband Doppler ultrasound mode to avoid major vessel injury when performing needle puncture. The wideband feature of this mode allows clear, real-time visualization of blood flow in renal parenchyma vessels due to reduction of blooming effects, which means vessels appear thicker than actual size on the image under ultrasound. Furthermore, we used an 8.5F to 9.5F tract sheath because the renal calyx in pediatric patients is smaller than that in adults. Large tracts of 24F to 30F may injure the renal parenchyma, potentially causing severe bleeding, transfusion, and future decline of renal function. In the present era, personalized management including individualization of the tract size, endoscope type, and instrumentation is critical in optimizing treatment outcomes.

## Conclusion

We successfully performed ultra-minimally invasive endoscopic intrarenal surgery in a 2-year-old boy with bilateral cystine kidney stones of >2 cm. Ultrathin semi-rigid ureteroscopy can be performed without prestenting in children. Furthermore, super ultra-mini ECIRS under the Barts modified Valdivia position is efficient and safe for pediatric patients with multiple large kidney stones.

## Abbreviations Used

KUB: kidney, ureter, and bladder
PCNL: percutaneous nephrolithotomy
RIRS: retrograde intrarenal surgery
super ultra-mini ECIRS: super ultra-minimally invasive endoscopic combined with intrarenal surgery
super ultra-mini PCNL: super ultra-minimally invasive PCNL
SWL: extracorporeal shockwave lithotripsy
US: ultrasonography
